# Account of Diseases in an Eastern District in London, from the 20th of October to the 20th of November, 1811

**Published:** 1811-12

**Authors:** 


### *Account of Diseases in an Eastern District in London, from the 20th of October to the 20th of November, 1811.*

| | |
|---|---|
| ACUTE DISEASES. | |
| Typhus Mitior | 3 |
| Peripneumonia | 2 |
| Dysenteria | 6 |
| Cholera | 4 |
| Rheumatismus Acutus | 2 |
| CHRONIC DISEASES. | |
| Tussis | 10 |
| Dyspnœa | 5 |
| Tussis cum Dyspnœa | 9 |
| Hæmoptysis | 4 |
| Pleurodyne | 3 |
| Hydrothorax | 4 |
| Apoplexia | 1 |
| Paralysis | 2 |
| Diarrhœa | 9 |
| Hæmorrhagia Intestinalis | 3 |
| Amenorrhœa | 2 |
| Dysmenorrhœa | 4 |
| Dysuria | 6 |
| Rheumatismus Chronicus | 10 |
| PUERPERAL DISEASES. | |
| Ephemera | 5 |
| Dolor post partum | 6 |
| Dysuria | 5 |
| Diarrhœa | 7 |
| Menorrhagia lochialis | 3 |
| INFANTILE DISEASES. | |
| Scrofula | 3 |
| Tabes Mesenterica | 4 |
| Pertussis | 3 |
| Convulsio | 1 |

It is at this season of the year that our principal attention is called to those diseases which have their seat in the intestinal canal. Diarrhœa, dysentery, and cholera, make a principal figure in a list of diseases. These have by some been attributed to an unusually large quantity of fruit, and which, in the present season, has not been in its most perfect state. To whatever cause, however, they may be attributed, they have not appeared in their most malignant form. The first of these has been so mild as not, in many instances, to require medical attention, and might be considered rather as a salutary effort of nature, to throw off something offensive to the constitution, than as producing a distinct disease. Where the intestinal discharge has been long continued, and has been accompanied by mucous and bloody bloody stools, rather than by a large evacuation of fœces, the disease has assumed a more serious aspect. It is then generally accompanied by some febrile symptoms, and sometimes proves contagious. The seat of it is in the larger intestines, and it has been referred to a spasmodic stricture of the colon and a detention of hardened fœces. The cure has too often been attempted by the use of astringent remedies, which, instead of removing the cause, have rather aggravated the symptoms; whilst the free exhibition of cathartics has proved highly useful in the removal of large quantities of accumulated fœces.

The removal of this source of irritation has been followed by a considerable diminution of the number of stools; and those severe gripings, and that troublesome tenesmus, by which they have been attended, have been relieved.

